# Pharmacological effects of Astragalus polysaccharides in treating neurodegenerative diseases

**DOI:** 10.3389/fphar.2024.1449101

**Published:** 2024-08-02

**Authors:** Yuanshu Shi, Ping Ma

**Affiliations:** School of Basic Medical, Chengdu University of Traditional Chinese Medicine, Chengdu, Sichuan, China

**Keywords:** neurodegenerative diseases, Astragalus polysaccharide, Alzheimer’s disease, Parkinson’s disease, multiple sclerosis

## Abstract

*Astragalus membranaceus* widely used in traditional Chinese medicine, exhibits multiple pharmacological effects, including immune stimulation, antioxidation, hepatoprotection, diuresis, antidiabetes, anticancer, and expectorant properties. Its main bioactive compounds include flavonoids, triterpene saponins, and polysaccharides. Astragalus polysaccharides (APS), one of its primary bioactive components, have been shown to possess a variety of pharmacological activities, such as antioxidant, immunomodulatory, anti-inflammatory, antitumor, antidiabetic, antiviral, hepatoprotective, anti-atherosclerotic, hematopoietic, and neuroprotective effects. This review provides a comprehensive summary of the molecular mechanisms and therapeutic effects of APS in treating neurodegenerative diseases, including Alzheimer’s disease (AD), Parkinson’s disease (PD), and multiple sclerosis (MS). It discusses how APS improve insulin resistance, reduce blood glucose levels, enhance cognitive function, and reduce Aβ accumulation and neuronal apoptosis by modulating various pathways such as Nrf2, JAK/STAT, Toll, and IMD. For PD, APS protect neurons and stabilize mitochondrial function by inhibiting ROS production and promoting autophagy through the PI3K/AKT/mTOR pathway. APS also reduce oxidative stress and neurotoxicity induced by 6-hydroxydopamine, showcasing their neuroprotective effects. In MS, APS alleviate symptoms by suppressing T cell proliferation and reducing pro-inflammatory cytokine expression via the PD-1/PD-Ls pathway. APS promote myelin regeneration by activating the Sonic hedgehog signaling pathway and fostering the differentiation of neural stem cells into oligodendrocytes. This article emphasizes the significant antioxidant, anti-inflammatory, immunomodulatory, and neuroprotective pharmacological activities of APS, highlighting their potential as promising candidates for the treatment of neurodegenerative diseases.

## 1 Introduction

Neurodegenerative diseases (NDDs) are a global public health issue and one of the leading causes of death and disability worldwide (Ayeni et al., 2022). Neurodegenerative diseases are a heterogeneous group of disorders characterized primarily by the progressive loss and degeneration of neurons within the central nervous system (CNS) or peripheral nervous system (PNS). These diseases typically present with a gradual decline in cognitive, motor, sensory, and behavioral functions, ultimately leading to severe disability and death. Major neurodegenerative diseases include Parkinson’s disease, Alzheimer’s disease, amyotrophic lateral sclerosis, and Huntington’s disease (Xing and Xu, 2021). Traditional Chinese medicine (TCM) has emerged as a promising approach, offering potential for the treatment of neurodegenerative diseases (Du et al., 2022).

**FIGURE 1:**
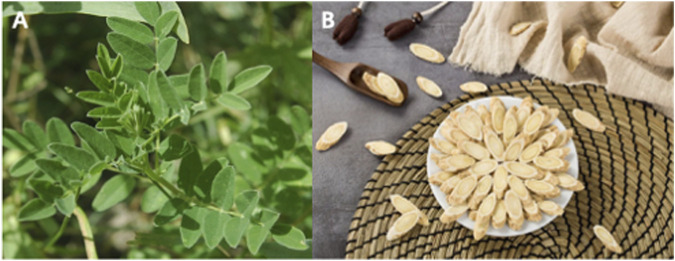
The aerial tissues **(A)** and commercial presentation **(B)** of Astragalus membranaceus.

*AM* (Figure 1) mainly consists of two varieties, A. *membranaceus* (Fisch.) Bunge var. Mongholicus (Bunge) P.K. Hsiao and A. *membranaceus* (Fisch.) Bunge var. (Hu et al., 2022). It is one of the most widely used traditional Chinese herbs, first recorded in the " Shen Nong’s Herbal Classic " (Wang et al., 2023) *Astragalus membranaceus* can be used as an immune stimulant, tonic, antioxidant, hepatoprotective agent, diuretic, antidiabetic agent, anticancer agent, and expectorant (Fu et al., 2014). The main active compounds obtained from *AM* include flavonoids, triterpene saponins, and polysaccharides, which exhibit a wide range of biological activities and pharmacological effects (Peng et al., 2023). Polysaccharides are natural macromolecular polymers composed of ten or more monosaccharides linked by glycosidic bonds to form linear or branched chains. They are widely distributed in nature and have attracted significant attention due to their diverse biological activities (Hou et al., 2020).

APS is one of the main bioactive components of *AM*, characterized by complex and diverse structures and multiple pharmacological activities, such as antioxidant effects (Niu et al., 2011). The main components of APS include heteropolysaccharides, neutral polysaccharides, glucans, and acidic polysaccharides, which are primarily linked by 1→4-glucosidic bonds (WANG, 2017). It has been demonstrated that APS possess various biological activities, including immunomodulatory, antioxidant, antitumor, antidiabetic, antiviral, hepatoprotective, anti-inflammatory, anti-atherosclerotic, hematopoietic, and neuroprotective effects (Chen et al., 2023). These findings not only indicate the therapeutic potential of APS in various diseases but also highlight their potential role in the development of new drugs.

This review comprehensively summarizes the latest advances in the molecular mechanisms and therapeutic effects of APS in the treatment of neurodegenerative diseases, enhancing the understanding of its therapeutic potential and recommending it as a candidate drug for the treatment of neurodegenerative diseases. These findings are of great significance for the development of effective drugs in the field of neurodegenerative disease treatment.

## 2 Overview of Astragalus membranaceus

AM is a leguminous plant widely distributed in China, Mongolia, and Korea, mainly growing in temperate and subtropical regions below 2000 m above sea level. *AM* is highly adaptable and usually grows on hillsides, grasslands, and forest edges (Auyeung et al., 2016). *AM* is a perennial herbaceous plant, about 50–150 cm tall, with a stout, cylindrical root covered with a yellow-brown outer skin and yellow-white inner part. The stem is upright, and the leaves are pinnately compound, with leaflets ranging from elliptical to lanceolate. The inflorescence is a raceme, usually with light yellow flowers, and the fruit is a pod. *AM*, as a traditional Chinese medicine, has been used for over 2000 years, first recorded in the “Shennong’s Classic of Materia Medica.” The primary medicinal part is the root, which has functions such as boosting qi to lift yang, benefiting the protective qi to consolidate the exterior, promoting diuresis to alleviate edema, and detoxifying to promote tissue regeneration. *AM* contains abundant chemical components, including flavonoids, saponins, polysaccharides, amino acids, and trace elements, which endow it with various pharmacological effects, such as immunomodulatory, antioxidant, anti-inflammatory, and anti-fatigue effects (Hou et al., 2023). To date, approximately 404 chemical constituents have been isolated and identified from Astragalus membranaceus, including saponins, flavonoids, phenylpropanoids, alkaloids, and other chemical compounds. APS can be further classified into water-soluble α-(1–4) (Fu et al., 2014; Xing and Xu, 2021; Ayeni et al., 2022; Du et al., 2022; Hu et al., 2022; Wang et al., 2023) glucan and water-insoluble α-(1–4) glucan (Liu et al., 2024).*AM* is widely used to enhance immunity, delay aging, lower blood sugar, protect the cardiovascular system, and fight tumors (Wang et al., 2019; Dong et al., 2023).

## 3 General biological activities of APS

**FIGURE 2:**
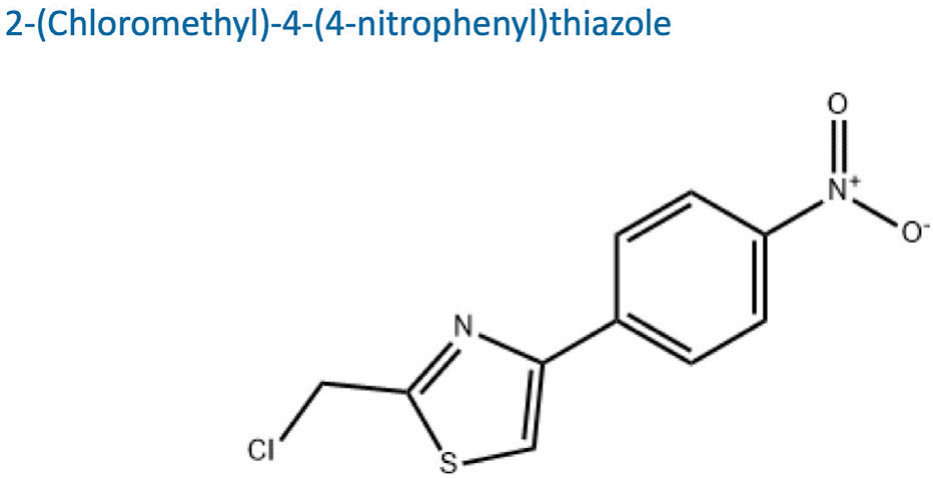
Chemical structural formula of APS.

**FIGURE 3:**
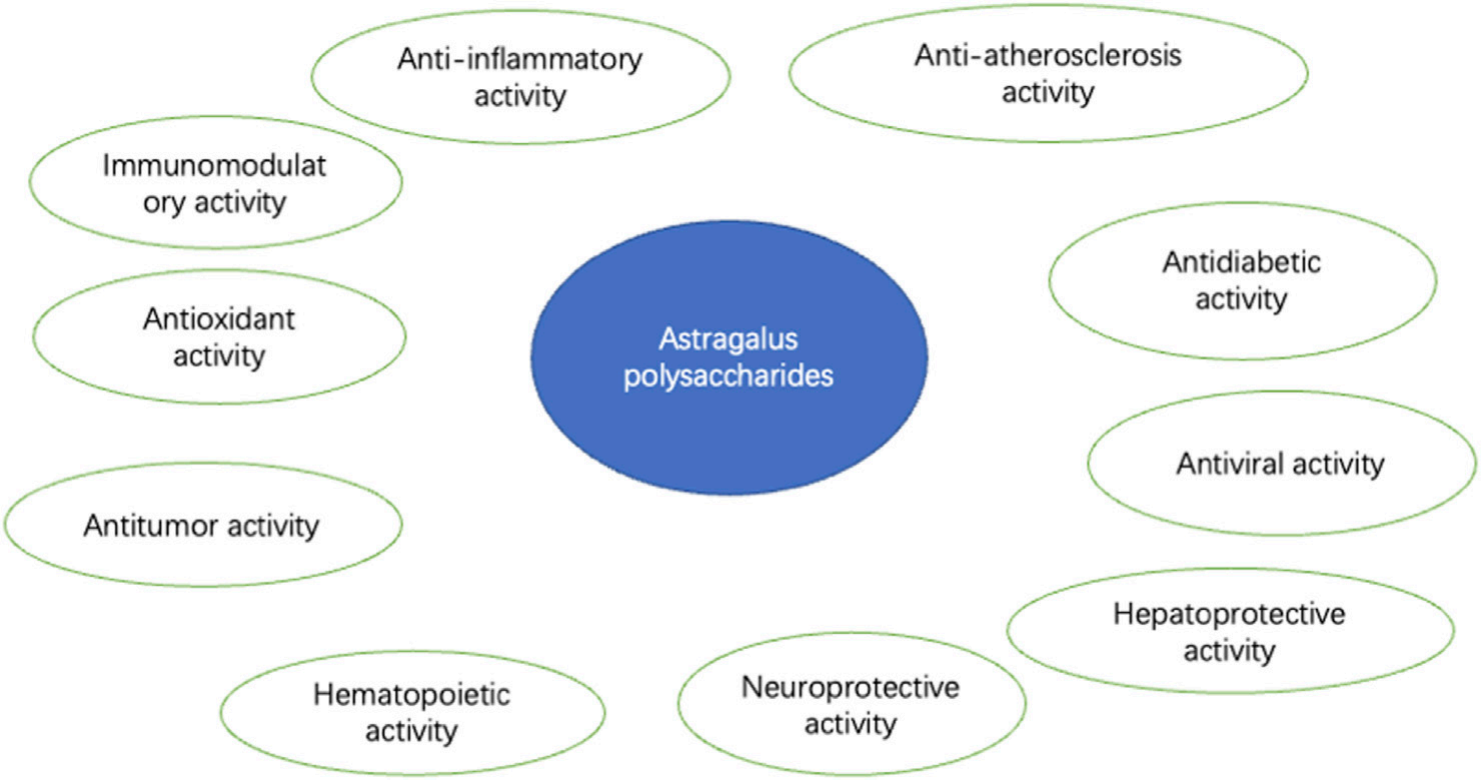
General pharmacological activities of APS.

APS(molecular formula: C10H7ClN2O2S, relative molecular mass:254.7) (Figure 2), is a bioactive component extracted from *AM*, appearing as a white or slightly yellowish powder. APS is primarily composed of fructose, rhamnose, arabinose, hexuronic acid, glucose, galacturonic acid, and glucuronic acid (Ren et al., 2023). APS is soluble in water but insoluble in alcohol and has good hygroscopicity (LI et al., 2009). Polysaccharides from Astragalus membranaceus are commonly extracted using methods such as cold water extraction, enzyme-assisted extraction, homogenization-assisted negative pressure cavitation extraction, and pressurized liquid extraction. The constituents of APS contain various monosaccharide residues and impurities. Current studies indicate that purification and separation methods may more or less affect the activity of the polysaccharides. A common method employed is column chromatography (Tang and Huang, 2022). APS exhibits a variety of biological activities (Figure 3), such as immunomodulatory, antioxidant, antitumor, antidiabetic, antiviral, hepatoprotective, anti-inflammatory, anti-atherosclerotic, hematopoietic, and neuroprotective effects.

APS can regulate the activity of various immune cells, including macrophages, natural killer cells, dendritic cells, T lymphocytes, B lymphocytes, and microglial cells. APS can induce these immune cells to produce various cytokines and chemokines, thereby enhancing the immune response (Li et al., 2022). APS can inhibit the proliferation, migration, and invasion of tumor cells. Liu et al. found that APS-induced cell cycle arrest at the G0/G1 phase in bladder cancer UM-UC-3 cells through inhibition of the JAK2/STAT3 pathway, thereby suppressing cell proliferation (Liu HW. et al., 2023). APS also exhibits antifibrotic effects by inhibiting the production of TGF-β1, thereby treating systemic sclerosis fibrotic diseases in a murine model induced by bleomycin (Hao et al., 2015). Diabetes is a serious chronic endocrine/metabolic disorder, and APS may reduce blood glucose and enhance insulin sensitivity in type 2 diabetes patients by alleviating endoplasmic reticulum stress. The mechanisms may include reducing ATF6 activation induced by endoplasmic reticulum stress, reversing intracellular ATF6 translocation, and inhibiting the high expression of certain protein phosphatases. These changes alleviate endoplasmic reticulum stress in the liver, thereby enhancing insulin sensitivity (Wang et al., 2009).

## 4 Pharmacokinetics of APS

Research on the pharmacokinetics of Astragalus Polysaccharides (APS) is relatively limited. APS is primarily absorbed through passive pathways *in vivo*, but its absorption efficiency is relatively low. The main reasons for this are its large molecular weight, low lipophilicity, and poor intestinal permeability (Chen et al., 2011). To enhance its bioavailability, researchers have explored improved drug delivery systems such as nanocarriers (Ghaffarian et al., 2016), which significantly increase the water solubility, stability, and biocompatibility of APS. Polysaccharides isolated from Astragalus are mostly extracted in the form of a white powder, and studies indicate that the relative molecular weight of APS ranges from 10 to 50 kDa (Jin et al., 2013). Additionally, moderate structural modifications can enhance the biological activity of APS, improving its therapeutic effects *in vivo*.

After oral or injectable administration, APS is primarily distributed in the liver, kidneys, spleen, lungs, and plasma, which are its main targets of action (Li et al., 2022). Some studies suggest that APS can improve the permeability of the blood-brain barrier, enhancing its distribution in the brain (Jia et al., 2022), and providing a potential advantage for its application in neurological diseases. APS is mainly metabolized by intestinal microbiota, with its metabolites including low molecular weight polysaccharides and monosaccharides, which can be further absorbed and utilized by the body. The main metabolic pathways include hydrolysis, glucuronidation, sulfation, and dehydrogenation.

Research indicates that APS has low toxicity at conventional doses, and no significant adverse reactions have been observed with long-term use (Chen et al., 2020). Early studies in Sprague-Dawley rats and Beagle dogs showed no histopathological or toxicological effects on body weight, organs, appetite, behavior, hematology, blood biochemistry, or urinalysis after prolonged oral administration (Yu et al., 2007). These studies of pharmacokinetic properties provide a scientific basis for the safety and efficacy of APS in clinical applications and offer important references for further optimizing its drug delivery systems and enhancing therapeutic effects.

## 5 Therapeutic effects and mechanisms of APS on neurodegenerative diseases

**FIGURE 4:**
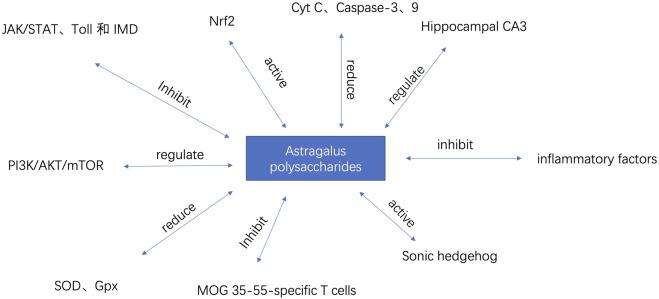
The regulatory mechanisms of APS in neurodegenerative diseases.

The prevalence and incidence of neurodegenerative diseases increase sharply with age (Checkoway et al., 2011). Major diseases include Alzheimer’s disease, Parkinson’s disease, and multiple sclerosis. Alzheimer’s disease is the most common form of dementia worldwide, accounting for 60%–80% of all dementia cases, affecting approximately 24 million people globally (Mayeux and Stern, 2012). Idiopathic Parkinson’s disease (PD) is the second most common neurodegenerative disease after AD. It is estimated that the prevalence of PD in the general population is 0.3%, about 1% in people over 60 years old, and about 3% in people over 80 years old, with an incidence of 8–18 per 100,000 person-years (Tanner and Goldman, 1996; Nussbaum and Ellis, 2003). The therapeutic mechanism of APS for neurodegenerative diseases is shown in Table 1. Part of the mechanism of action of APS is shown in Figure 4

**TABLE 1:** The regulatory effects and mechanisms of APS on neurodegenerative diseases.

Disease type	Model	Mechanism (pathway) and effect	Dose	References
	HFSTZ-induced APP-PS1 mice	Insulin levels and insulin resistance levels↓		Tanner and Goldman (1996)
	Aβ42-induced *Drosophila*	JAK/STAT, Toll and IMD pathways↓	3 mg/mL APS	Nussbaum and Ellis (2003)
AD	(AD) APP-PS1 mice	Nrf2↑, Keap1↓	Oral APS 200 mg/kg	Huang et al. (2017)
	AD rats	Cyt-C and Caspase-3,9↓	0.2g/0.4g/0.8 g/kg APS gavage	Li et al. (2023)
	AD mice	Hippocampal Aβ and IL-6↓	120 μg/mL	Qin et al. (2020)
	HSV-1 astrocytes	TLR3/NF-κB↑		(Qu et al.)
	PC12 cell	PI3K/AKT/mTOR pathway↓		Matheoud et al. (2016)
PD	6-Hydroxydopamine (6-OHDA) induced *C. elegans*	ROS↓, SOD, GPx↑, egl-1↓		Tan et al. (2020)
	PD-wistar rats	bFGF protein, IL-2, IL-6↓	1 g/kg/d	Pringsheim et al. (2014)
	EAE-induced C57 BL/6 mice	PD-1/PD-Ls↑	Oral 500mg/(kg·d)	Garg and Smith (2015)
MS	CPZ-induced demyelination model in mice	Sonic hedgehog signaling pathway activation		Sun et al. (2019)

### 5.1 Alzheimer’s disease (AD)

The pathogenesis of AD is complex, and current treatments can only improve cognitive function and delay the degenerative process, with significant limitations. Epidemiological studies have shown that type 2 diabetes (T2DM) increases the risk of AD. The effects of APS in improving insulin resistance and lowering blood glucose can reduce the risk of AD from the source. In experiments with HFSTZ AD mice, APS reduced glucose intolerance induced by HFSTZ, and the elevated insulin levels (hyperinsulinemia) and insulin resistance (increased HOMA-IR) induced by HFSTZ were alleviated by APS (Huang et al., 2017). Aging increases the risk factors for neurodegenerative diseases such as AD and PD. In anti-aging experiments with fruit flies, APS significantly alleviated intestinal homeostasis imbalance, improved sleep disorders, delayed the occurrence of AD-like phenotypes in Aβ-related AD fruit flies, and rescued aging-induced changes in the JAK/STAT, Toll, and IMD pathways (Li et al., 2023).

APS can increase the expression of Nrf2 in the nucleus of mouse cells, reduce its expression in the cytoplasm, and restore the expression levels of Keap1, SOD, GSH-Px, and MDA. APS can significantly improve the cognitive ability of APP/PS1 mice by activating the Nrf2 pathway, reducing cell apoptosis and Aβ accumulation, thereby improving the physiological function of AD mice (Qin et al., 2020). APS can reduce cell apoptosis by downregulating the expression of Cyt C and Caspase-3、9, thus protecting neurons (Qu et al.). Studies have shown that after injecting Aβ into AD mice, the learning and memory abilities of the mice significantly improved. Pathological examination revealed that the organ index of the brain increased, the water content and Evans blue content in the brain tissue decreased, and the neuronal structure in the CA3 region of the hippocampus was relatively normal, with abundant organelles, clear nuclear membranes, and normal mitochondrial morphology (Fei et al., 2015). This indicates that APS has pharmacological activity in improving Alzheimer’s disease. When astrocytes are continuously stimulated by stress factors, they can promote the occurrence and development of neurodegenerative diseases by releasing inflammatory factors and free radicals. APS can not only directly inhibit the aggregation of disease proteins but also reduce the damage of herpes simplex virus to astrocytes (Shi et al., 2014). In conclusion, APS can not only improve insulin resistance and lower blood glucose but also fundamentally improve AD through pathways such as JAK/STAT, Toll, IMD, and Nrf2.

### 5.2 Parkinson’s disease (PD)

Parkinson’s disease (PD) initially affects multiple neurons, with later stages or progression primarily involving damage to the central nervous system (Kalia and Lang, 2015). Mitochondrial dysfunction is one of the main mechanisms of Parkinson’s disease. Protecting and repairing damaged neurons and mitochondria is a major approach to preventing and treating Parkinson’s disease (Pickrell and Youle, 2015; Matheoud et al., 2016). Autophagy is an important intracellular energy metabolism and self-renewal mechanism in eukaryotic cells, closely related to the occurrence and development of Parkinson’s disease (Heras-Sandoval et al., 2014). APS can promote the formation of autophagosomes within cells, increase the conversion of LC3-I protein to LC3-II protein, downregulate the expression of pAKT and p-mTOR proteins, and upregulate the expression of PTEN protein, thereby exerting anti-PD activity. APS can promote autophagy in PC12 cells through the PI3K/AKT/mTOR pathway, thereby promoting cell proliferation and exerting anti-PD effects (Tan et al., 2020). Clinical and epidemiological survey evidence suggests that the onset and progression of PD are closely related to the aging process (Pringsheim et al., 2014). Levodopa is an important drug for treating Parkinson’s disease, but it has neurotoxicity. 6-Hydroxydopamine (6-OHDA) is a neurotoxin that can induce Parkinson’s disease by causing the degeneration of dopaminergic neurons. APS can alleviate oxidative stress by reducing the levels of reactive oxygen species and malondialdehyde, increasing the activities of superoxide dismutase (SOD) and glutathione peroxidase (Gpx), and reducing the expression of the pro-apoptotic gene *egl-1* in 6-OHDA-intoxicated *Caenorhabditis elegans*. APS can also increase acetylcholinesterase activity in 6-OHDA-induced models (Li et al., 2016). This study provides important evidence for the therapeutic potential of APS in neurodegenerative diseases. Research has shown that APS can reduce the number of rotations in Parkinson’s model rats, increase the content of tyrosine hydroxylase, reduce the expression of bFGF protein in the substantia nigra, and decrease the levels of inflammatory cytokines in brain tissue (Chen and Zhou, 2015). This suggests that APS may treat Parkinson’s disease by regulating the body’s immune function.

### 5.3 Multiple Sclerosis (MS)

Multiple sclerosis (MS) is an acquired disabling neurological disease affecting approximately 2.3 million people worldwide (World Health Organization, 2008). MS is an autoimmune inflammatory disease of the central nervous system characterized by demyelination and varying degrees of axonal loss (Garg and Smith, 2015). Its clinical manifestations include severe neurological dysfunction, such as ataxia, visual impairment, and aphasia, eventually leading to paralysis and even death (Dendrou and Fugger, 2017). Studies have found that APS can effectively alleviate EAE by inhibiting the proliferation of MOG 35-55-specific T cells and reducing the expression of pro-inflammatory cytokines. This is mediated by upregulating the PD-1/PD-Ls signaling pathway (Sun et al., 2019). This indicates that APS may have the potential to develop innovative drugs for treating multiple sclerosis. Currently, immunosuppressants and immunomodulators are primarily used to treat MS, which can alleviate symptoms and delay relapses but cannot effectively restore patients’ neurological functions (Wingerchuk and Carter, 2014). Current clinical research shows that the degree of myelin regeneration directly affects the recovery of neurological function and the improvement of quality of life. Therefore, promoting myelin regeneration is an important strategy for treating MS. In a demyelination mouse model, APS was found to downregulate the expression of Nestin and GFAP molecules at both transcriptional and protein levels, promote the expression of MBP molecules, and upregulate the expression of NeuN molecules, indicating that APS can promote the differentiation of neural stem cells into oligodendrocytes *in vivo*. This may be one of the mechanisms by which APS promotes myelin regeneration. Additionally, the study found that APS can activate the Sonic hedgehog signaling pathway both *in vivo* and *in vitro*, which may be the molecular mechanism by which APS regulates the differentiation of neural stem cells into oligodendrocytes (Ye et al., 2021).

## 6 Conclusion and future directions

APS are natural plant extracts that have been widely recognized for their pharmacological effects in neurodegenerative diseases. As discussed, APS exert neuroprotective effects through various biological pathways, including antioxidant, anti-inflammatory, immunomodulatory, anti-apoptotic, and neuroregenerative activities. Their specific mechanisms involve the regulation of multiple cell signaling pathways, such as PI3K/AKT/mTOR, JAK/STAT, and Nrf2, demonstrating significant therapeutic potential.

Despite the substantial progress made in APS research for neurodegenerative diseases, future studies need to delve deeper into several key areas. First, it is necessary to further elucidate the molecular targets and mechanisms of action of APS. Current research on the molecular targets and mechanisms of APS is predominantly focused on tumors and fibrosis (Bing et al., 2022; Liu WZ. et al., 2023; Sun et al., 2023), with limited studies in the field of neurodegenerative diseases. Comprehensive analysis using multi-omics technologies should be conducted to fully unravel these mechanisms and provide a theoretical basis. Second, most current research is still at the animal or cell experimental stage, lacking systematic clinical studies. Large-scale, multicenter randomized controlled clinical trials and long-term follow-up studies should be conducted to verify the efficacy and safety of APS in patients. Moreover, Astragalus polysaccharide solutions contain a considerable amount of impurities (Li et al., 2019), making the separation and purification process complex and time-consuming. Therefore, the purification and separation of natural products have always been a challenging aspect of the extraction process (Tang and Huang, 2022). Optimizing the extraction and purification techniques for Astragalus polysaccharides, as well as developing new drug delivery methods and formulation types, can improve their bioavailability and patient compliance. Personalized treatment is also an important direction for the future. By identifying biomarkers and applying precision medicine, individualized treatment plans can be developed to maximize efficacy. Lastly, as an extract of Astragalus, APS exhibits minimal toxic side effects. While exerting its pharmacological effects, precise dosage control is essential, requiring further in-depth investigation by researchers. Exploring the combined application of APS with existing drugs and integrated traditional Chinese and Western medicine treatment strategies will help enhance overall therapeutic effects and reduce side effects. Through these comprehensive studies, APS is expected to play a greater role in the treatment of neurodegenerative diseases.

## Figures and Tables

**Table:** 

**AD**	Alzheimer’s disease
**APS**	Astragalus Polysaccharides
**BBB**	Blood-Brain Barrier
**Bcl-2**	B-cell lymphoma 2
**CNS**	Central Nervous System
**CRP**	C-reactive protein
**CAT**	Catalase
**EBV**	Epstein-Barr Virus
**NDDs**	Neurodegenerative diseases
**EAE**	Experimental Autoimmune Encephalomyelitis
**GSH**	Glutathione
**GSH-Px**	Glutathione Peroxidase
**HFSTZ**	High-Fat Diet and Streptozotocin
**IL**	Interleukin
**JAK/STAT**	Janus Kinase/Signal Transducer and Activator of Transcription
**LDH**	Lactate Dehydrogenase
**MAPK**	Mitogen-Activated Protein Kinase
**MBP**	Myelin Basic Protein
**MDA**	Malondialdehyde
**MS**	Multiple Sclerosis
**mTOR**	Mammalian Target of Rapamycin
**NF-κB**	Nuclear Factor Kappa-Light-Chain-Enhancer of Activated B Cells
**NGF**	Nerve Growth Factor
**NLRP3**	NOD-Like Receptor Protein 3
**NO**	Nitric Oxide
**Nrf2**	Nuclear Factor Erythroid 2-Related Factor 2
**PD**	Parkinson’s Disease
**PS1**	Presenilin 1
**PI3K**	Phosphatidylinositol-3-Kinase
**PD-1**	Programmed Death-1
**PD-Ls**	Programmed Death Ligands
**PTEN**	Phosphatase and Tensin Homolog
**ROS**	Reactive Oxygen Species
**SOD**	Superoxide Dismutase
**T2DM**	Type 2 Diabetes Mellitus
**TLR**	Toll-Like Receptor
**TNF-α**	Tumor Necrosis Factor Alpha
**TrkA**	Tropomyosin Receptor Kinase A
